# Fluorescence-Based Multimodal DNA Logic Gates

**DOI:** 10.3390/nano14141185

**Published:** 2024-07-12

**Authors:** Chamika Harshani Algama, Jamil Basir, Kalani M. Wijesinghe, Soma Dhakal

**Affiliations:** Department of Chemistry, Virginia Commonwealth University, Richmond, VA 23284, USA

**Keywords:** DNA logic gates, four-way DNA, fluorophore, quencher, multimodal

## Abstract

The use of DNA structures in creating multimodal logic gates bears high potential for building molecular devices and computation systems. However, due to the complex designs or complicated working principles, the implementation of DNA logic gates within molecular devices and circuits is still quite limited. Here, we designed simple four-way DNA logic gates that can serve as multimodal platforms for simple to complex operations. Using the proximity quenching of the fluorophore–quencher pair in combination with the toehold-mediated strand displacement (TMSD) strategy, we have successfully demonstrated that the fluorescence output, which is a result of gate opening, solely relies on the oligonucleotide(s) input. We further demonstrated that this strategy can be used to create multimodal (tunable displacement initiation sites on the four-way platform) logic gates including YES, AND, OR, and the combinations thereof. The four-way DNA logic gates developed here bear high promise for building biological computers and next-generation smart molecular circuits with biosensing capabilities.

## 1. Introduction

Molecular logic gates have emerged as novel computation systems that utilize distinct recognition elements such as DNA, RNA, and proteins as well as biochemical pathways for myriad applications in various fields including data storage, bioinformatics, medical diagnostics, and bio- and nanotechnology [1]. Logic gates utilize predetermined designs and principles that determine whether a general outcome would be TRUE or FALSE for a given input(s). For example, logic gate elements can be incorporated into logical relationships to determine predictable outcomes and even solve practical problems [2]. Binary logic gates, also called Boolean gates, are the most common types and share the same main framework for all processing devices such as computers [3], which work based on the binary digits 0 and 1 [4]. As mentioned before, these gates are associated with a wide range of applications such as electronic, optical, and mechanical, including recent developments in biological systems. These developments have been possible by combining Boolean functions to create unique, predictive, and customizable circuits and nanodevices such as YES, AND, OR, NOR, NOT, NAND, etc., which follow a set of algorithms and generate a response based on the input conditions [5].

Recently, molecular logic gates have been considered promising alternatives to traditional silicon-based chips due to their advantages and applications including ion detection [6,7], heavy metal analysis [8,9], food safety testing [10,11], cell imaging [12], pharmaceutical analysis [13,14], and disease diagnosis [15,16]. Some notable examples include chemical receptors for simple and sensitive detection of ions in aqueous media. For example, an “INHIBIT” gate has been demonstrated using selective receptors for F^−^ and AcO^−^ ions using the colorimetric method [17]. Other notable examples include the “AND” gate for the detection of Mn^7+^ using a fluorescent probe, where Mn^7+^ and L-ascorbic acids are inputs, which provides different fluorescence outputs [18]. Similarly, heavy metal analysis has been demonstrated using several fluorescence-based logic gate implementations. For example, Cd^2+^ detection using AND, OR, NOR, INHIBIT, and NAND gates [10], Ni^2+^ detection via “YES” gate in combination with DNA template silver nanoclusters, and Ni^2+^ and Hg^2+^ detection using an “INHIBIT” gate [19]. The detection of Pb^2+^ and Hg^2+^ has also been demonstrated using the “INHIBIT” gate combined with surface-enhanced Raman scattering (SERS) [8]. A cascade of the “INHIBIT-OR” gate has been developed and successfully utilized for the detection of miRNA biomarkers [miR-122 and miR-Let-7a], and a multi-input logic gate has been designed to distinguish different miRNA expression patterns of specific cancer cells [15,20].

While these new initiatives are emerging, several reports have been published in recent years focusing on DNA-based logic gates with applications in interdisciplinary research aspects, primarily due to the low cost of DNA, easy synthesis, and superior biocompatibility [16,21,22]. These efforts have demonstrated different ways of constructing logic gates including DNA self-assembly [23], strand displacement [24], and DNAzymes [25]. Since the first DNA computing was proposed by Adelman in 1994 to solve the Hamiltonian path problem [26], extensive research on DNA logic gates has been conducted in biocomputing [27]. Several reports have leveraged switchable chemical systems to create molecules responding to external physical and chemical signals [28]. Notable examples include Boolean logic operations such as AND, OR, XOR, NOR, NAND, INHIBT, and XNOR incorporated into the DNA platforms, and they have shown tremendous potential in biosensing [29]. In these molecular gates, DNA input and output are provided in the form of encoded strands that are involved in biochemical reactions [30]. These gates were developed for biosensing applications using targets as inputs and the signal readout as outputs. DNA-based logic gate sensors are also intended for smart therapeutic applications because they are biocompatible and can be designed to delay cellular degradation [31]. In addition, DNA logic gates showed great potential for quantitative measurements [29,32]. An aptamer-based 3D logic operation as an “AND” gate was created to identify overexpressed cancer markers on the surface of cancer cell markers [33]. Similarly, various DNA logic gates systems have been constructed to detect different types of cancer by evaluating the miRNA expression levels [34]. For example, multiplex detection of miRNA for disease diagnostics has been developed using a cascading strand displacement DNA logic system [35]. DNA logical operations were also explored successfully in combination with G quadruplex which enhances the versatile potential of DNA logic gates [36]. A series of DNA logic gates (YES, AND, OR) were prepared using G quadruplex structures, which show specific binding capacity with the targets, as well as varying the number of inputs for a gate [37,38]. With the development of DNA logic gates, new sophisticated tools and mechanisms have been explored in several interdisciplinary research fields [39]. DNA molecular logical circuits using AND, NAND, OR, and NOR gates were designed to be used as luminescent lanthanide complexes to replace traditional fluorescent markers [40]. The development of a molecular keypad lock was reported for the molecular security and protection of biological information at the molecular scale, where the basic mechanism includes the presence of fluorescence signal only in response to correct sequences of three input signals [41]. Interestingly, the DNA logic gates can be expanded to build logic circuits for nonarithmetic information processing [42]. In summary, DNA logic gates have been used for various applications [39,43], including the next generation of advanced technologies and innovations such as nanorobots [44,45], intelligent DNA molecular machines [46,47], and nucleic acid detection by smartphone imaging [48]. However, there is still a need for simple-to-operate and multimodal DNA logic gates to further the potential of biocomputing systems.

In this manuscript, we leveraged four-way DNA junctions that can generate predetermined outcomes based on the oligonucleotide input to create multimodal logic gates. The junction is composed of four ssDNA, to which a fluorophore (Cy3) and a quencher (BHQ_2_) were incorporated in a way that the fluorescence remains quenched initially and the fluorescence output relies on the oligonucleotide input(s). In this strategy, the input strand initiates strand displacement via the process called toehold-mediated strand displacement (TMSD) [49,50], leading to the collapse of the junction and thereby freeing up the fluorophore and quencher strands and restoring the fluorescence. Several gates (YES, AND, OR, and a combination thereof) were tested. It is important to note that the TMSD strategy, as used here, is very useful, as it enables enzyme-free operations of gates, keeps the cost low, and provides a high level of design flexibility [27,51,52]. Using this approach and by designing gates that require either one or more inputs, we successfully demonstrated YES, AND, OR, simultaneous operation (SO), and cascading gates. Using one of the logic gate designs in a proof-of-concept experiment, we also showed that the gates can be reversible. The multimodal nature of the gates is also verified using TMSD initiated at the different arms of the four-way junction. Further, the time a logic gate takes from the input to the output step is rapid, which is a highly desirable feature of any logic gate. Therefore, these multimodal logic gates with many advantages outlined above will be of great use in the development of sensors, actuators, biocomputing, and beyond.

## 2. Materials and Methods

Chemical reagents: Protocatechuic acid (PCA), Protocatechuate 3,4-dioxygenase (PCD), 6-hydroxy-2,5,7,8-tetramethyl chroman-2-carboxylic acid (Trolox), magnesium chloride hexahydrate, and PCD was prepared by dissolving it in a PCD stock buffer (pH 8.0) consisting of 100 mM Tris-HCl, 50 mM KCl, 1mM EDTA, and 50% glycerol. All ssDNA sequences were purchased from Integrated DNA Technologies (IDT Inc., Coralville, IA, USA). The buffer used was 1 × TAE-Mg buffer (pH 7.4) (40 mM Tris, 20 mM acetic acid, 1 mM EDTA, and 10 mM Mg^2+^).

DNA sequences and logic gate designs: All of the modified and unmodified DNA oligonucleotides were dissolved in sterilized water to a final concentration of 100 μM and stored at −20 °C until needed. The four-way DNA junctions related to each logic gate were custom-designed based on the DNA/DNA hybridization principle. The list of sequences used to prepare the - four-way junctions is provided in Supplementary Table S1 and a schematic of the four-way DNA junction with the sequence detail for one of the junctions used is provided in Supplementary Figure S1. Briefly, the four-way DNA junction is composed of four oligonucleotides, one of which is labeled with a fluorophore (Cy3) and another is labeled with a quencher (BHQ2). Both the YES and OR gates consist of 16 nts in the labeled strands and the AND gate consists of 24 nts. These lengths were optimized for optimal stability of the junctions and at the same time feasible for strand displacement. Based on our experience, the annealed four-way constructs stay intact for at least two weeks after annealing when stored at 4 °C. The optimized arm lengths of labeled strands for the YES and OR gates consist of 8 bp, while the AND gate includes 16 bp. One of the unlabeled strands in the YES gate and both unlabeled strands of the AND and OR gates consist of a toehold of 8 nts.

Preparation of logic gates and fluorescence measurements: The logic gates were assembled by thermal annealing of 1 μM of each strand on a thermal cycler, as described in our previous publications [53,54]. The buffer used was 1 × TAE-Mg buffer (pH 7.4; 40 mM Tris, 20 mM acetic acid, 1 mM EDTA, and 10 mM Mg^2+^). Thermally annealed four-way DNA junctions (30 nM final) were mixed with 10 mM MgCl_2_, 1× TAE buffer, and double sterile water for a total of 200 µL solution to maintain the stability of the junction. As Cy3 fluorophores are prone to oxidative damage, an oxygen scavenging system (OSS) made of PCA (10 mM), Trolox (4 mM), and PCD (100 nM) was used to inhibit the photobleaching and photoblinking [55,56]. Input strands (150 nM) were subsequently added and changes in fluorescence intensity were recorded as a function of time on a Denovix fluorescence spectrophotometer (DS-11 FX+). The stoichiometry between the DNA junctions and input concentrations was kept at 1:5 in all experiments. Upon addition of the input(s), the samples were excited with a green excitation at 525 nm, and the fluorescence emission intensity was measured for 5 min at intervals of 30 s. The fluorescence output of the blank solution containing no input(s) was used for background correction. All fluorescence measurements were taken in triplicates at room temperature, background corrected, and averaged. The fluorescence intensity (RFU) in the absence and presence of the inputs was plotted against time (min). For the cascading circuits, the fluorescence intensity recorded at 5 min after adding the input was plotted against the type of unit.

## 3. Results and Discussion

**Figure 1:**
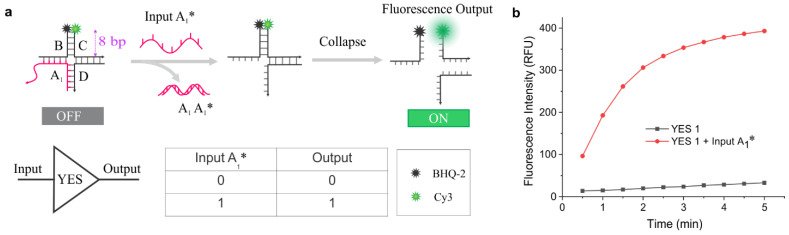
Design and characterization of YES gate: (**a**) Schematic illustration of the working principle of the YES-1 gate and the truth table with and without input. (**b**) The fluorescence signal recorded in the absence (control) and presence of input (A_1_*) over time. The gates were annealed using corresponding strands each at 1 µM final concentration, and the working concentration of each gate was 30 nM. All measurements were performed in triplicates. The corrected fluorescence (F − F_0_) was calculated by subtracting the fluorescence of the sample (F) from the fluorescence of the control (F_0_).

The YES gate (YES-1) is considered the simplest logic operation, and it is also known as the ‘identity’ function [57]. Here, unlike routine single design, we generated three designs of the gate (YES-1, YES-2, and YES-3) by changing the toehold position in the DNA four-way construct. The main purpose of generating these alternative designs is to test the multimodal capability of the gate and to confirm whether the length, sequence composition, and position of the toehold affect the operation of the gates. These gates were designed to have eight base pairs (bp) in the labeled arm so that the fluorophore and the quencher strands get separated out easily when input is added. Each strand is hybridized into two separate adjacent strands, which form the four-way junction (Figure 1a). The assembly of gates was verified using an electrophoretic mobility shift assay (Supplementary Figure S2). Figure 1b depicts what happens to the fluorescence signal in the absence and presence of the ssDNA input (A_1_^*^). While there was a very low fluorescence signal in the absence of input, the fluorescence signal increased significantly and reached a plateau at around 5 min when an input (A_1_^*^) was added. At the plateau, the fluorescence signal was nearly fivefold greater than the fluorescence recorded for the control construct without the input. We also noted a slight increase in the background fluorescence over time, which may be the result of some level of dissociation of the gate due to its short arms (8-bp). The standard deviation analysis of the gate showed less than 1% error in the mean fluorescence when the signal reached near saturation (Supplementary Table S2). Overall, the results demonstrate the successful assembly and operation of the gate. Inspired by this, we designed two more alternative YES gates, called YES-2 and YES-3 (Supplementary Figures S3 and S4), to demonstrate the multimodal capability of the four-way platform. As expected, these alternative designs showed consistent results with YES-1, demonstrating that the design is multimodal.

Another important parameter of the logic gates is their recyclability as the regeneration of the circuits is intended not only to save time and cost but also to make them more amenable for sustained use. However, the vast majority of traditional logic gates that are based on TMSD strategies are unidirectional. In this study, we considered this limitation and designed gates that can be regenerated by reversing the steps, which we demonstrated using the YES-1 gate as a proof-of-concept platform (Supplementary Figure S5).

**Figure 2:**
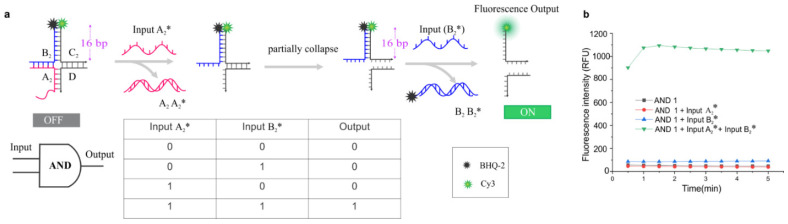
Design and characterization of AND gate: (**a**) Schematic illustration of the working principle of the AND-1 gate and the truth table with and without input(s). (**b**) Time-dependent fluorescence signal in the absence (control) and the presence of inputs (A_2_* and B_2_*). The working concentration of the gate was 30 nM.

After the successful designing and operation of the YES gates, we designed and characterized AND gates where two sequential inputs are needed to yield the fluorescence signal. The AND-1 gate was designed to have a 16 bp double-stranded arm when Cy3 and BHQ2labeled strands hybridize to one another. Due to the higher stability of the construct, the background fluorescence is expected to be low for this design. Additionally, the reason for increasing the length of the arm in the AND gate as compared with the YES gate was to prevent separation of the fluorophore and quencher at the first-step addition of input (Figure 2). The gate was designed such a way that the first input leads to a partial collapse of the junction due to the first toehold displacement step, exposing a second toehold. Due to the sufficient stability of the 16 bp double-stranded region formed by the labeled strands, the junction would not fully dissociate until the second TMSD step. Therefore, both inputs (A_2_^*^) and (B_2_^*^) must be present to induce dissociation of the AND-1 gate, resulting in fluorescence (Figure 2b).

As expected, the results show that there was almost no Cy3 fluorescence and that either input (A_2_^*^) or input (B_2_^*^) did not yield a significant change in fluorescence. However, when both inputs were added, it yielded a much higher fluorescence (~10 fold) than that obtained from the input (A_2_^*^) or input (B_2_^*^) alone. Similar to the YES-1 gate, additional experiments were carried out using AND-2 and AND-3 gates to determine if the position of the toehold matters in terms of the fluorescence output. The analysis showed that these alternative designs showed consistent results with AND-1 (Supplementary Figures S6 and S7). Also, it is important to note that the increased stability of this gate due to the 16 bp arm between the labeled strands kept the background low when compared with the YES-1 shown in Figure 1.

**Figure 3:**
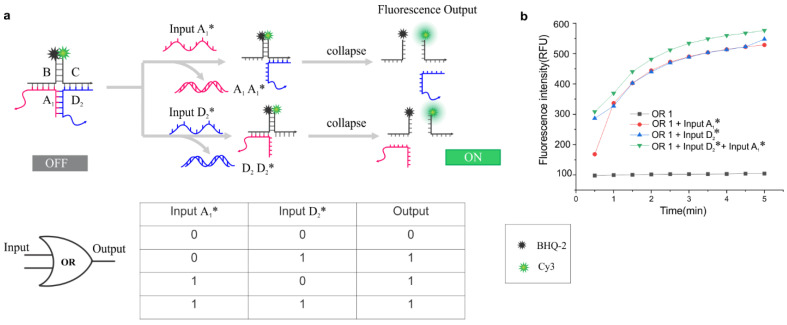
Design and characterization of OR gate: (**a**) Schematic illustration of the working principle of the OR-1 gate and the truth table with and without input. (**b**) Time-dependent fluorescence signal in the absence (control) and the presence of inputs (A_1_* and D_2_*). The working concentration of the gate was 30 nM.

The OR gate (OR-1) was designed using a similar strategy to YES and AND gates, except that this gate contains two possible inputs that can be used independently or simultaneously. In this gate, the input (A_1_*) and (D_2_*) are complementary to the strands A_1_ and D_2_, respectively. Each of these input strands was designed to bind to corresponding toeholds and initiate strand displacement so that the gate could collapse. As expected, we observed a sharp increase in the fluorescence intensity soon after adding the single and double inputs (Figure 3b). Regardless of which of the two inputs was added, the resultant fluorescence was similar. In fact, the fluorescence with any of the input(s) was about three-fold higher than that of the same gate without the inputs. In addition, the fluorescence output without input remained unchanged throughout the experiment, demonstrating the high stability of the logic gate. Similarly to the YES and AND gates, we also tested alternative designs of the OR gate using OR-2 and OR-3, demonstrating that all designs provide similar results (Supplementary Figures S8 and S9).

**Figure 4:**
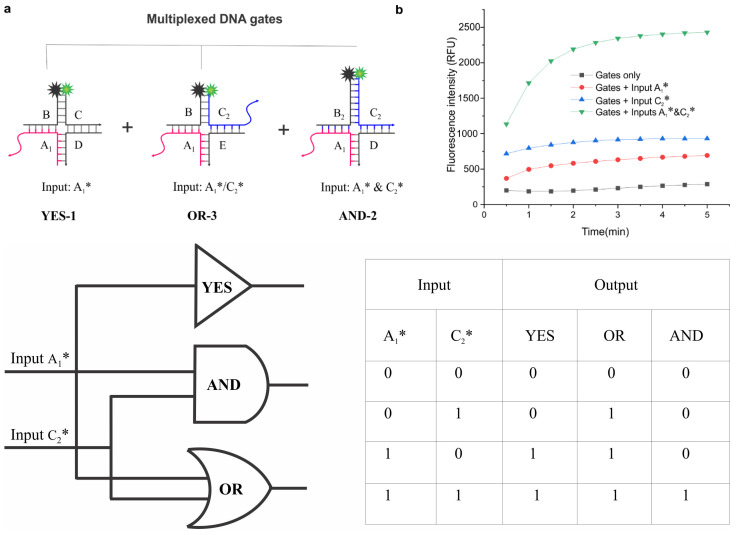
Design and characterization of simultaneous operation (SO) gate: (**a**) Schematic illustration of simultaneous operation and the truth table with and without inputs. (**b**) Monitoring the fluorescence signal in the absence (background) and the presence of inputs (A_1_* & C_2_*) over time. The working concentration of each gate was 30 nM.

The combination of simple logic gates can create more robust computation systems to perform complex logical operations. One scenario where such a gate would be useful is in the study of multiple targets, where the targets can be detected sequentially in one solution [58,59]. Therefore, we generated simultaneous operation (SO) gates by combining YES, AND, and OR gates, specifically the YES-1, AND-2, and OR-3 gates (Figure 4). The results show that the fluorescence intensity in the SO gate relies on whether one, two, or all three types of gates opened, which ultimately depends on the inputs used. Regardless of the relatively complex mixture of gates used in these experiments, the gates-only data showed a low and stable background ensuring leak-free gates. Overall, this platform can be highly advantageous in creating gates for multiplexed operations.

**Figure 5:**
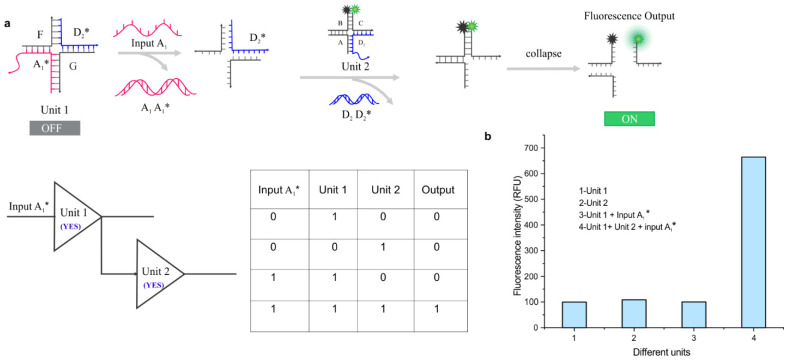
Design and characterization of cascading gate: (**a**) Schematic illustration of the working principle of cascading circuits in which two YES gates are combined. (**b**) Fluorescence outcomes when using various combinations of units with and without input, demonstrating both the units and input are needed for output. The working concentration of each gate was 30 nM.

The construction of more sophisticated gates such as cascading circuits is possible by combining more than one similar or different type of logic gate [60]. Cascading circuits utilize multi-input, in which the output of the first gate is utilized as an input for the second gate and so on, which eliminates the need for the traditional multilevel cascade operations. Here, we designed and tested a cascading circuit by combining two YES gates that act as sequential units (which we call Unit 1 and Unit 2), and the system was thoroughly evaluated to confirm the operation of the units in an input-dependent manner (Figure 5). As shown in Figure 5b, the fluorescence output for Unit 1, Unit 2, and Unit 1 + Input A_1_* all showed similar fluorescence signal, which is at least sixfold lower than the signal achieved when both units and Input A_1_* are present.

## 4. Conclusions

The use of DNA in constructing logic gates is particularly promising due to its high-density storage and potential for parallel computing. Therefore, the DNA logic gates can offer many unprecedented applications. The literature has provided several examples of DNA logic gates that are responsive to different logical relations; however, most of them are either unidirectional and need multistep inputs or utilize complicated designs. In this manuscript, we presented simple four-way DNA constructs that can be easily customized to develop multimodal gates highly responsive to DNA inputs. We tested various modes of logical operations of these gates, including the simple YES, AND, and OR gates and more advanced SO and cascading gates, and showed that the collapsed gates can also be reversed by reversing the steps. It may be possible to design multiplier circuits employing the DNA four-way logic gates using the strategies reported before [61]. In addition, using the four-way DNA systems, the development of more complex systems such as the feedback logic gates is possible. However, it is important to note that it will require careful designing of gates to serve the purpose. The demonstrated input specificity, selectivity, low background, and simple operation are the major advantages of these gates. The simultaneous operation and cascading nature of circuits with very high specificity bring a novel concept for the development of circuits useful for biocomputing systems.

Another key advantage of the DNA logic gates developed here is that they are enzyme-free. One potential challenge associated with the DNA-based logic gates is that the toehold length needs to be optimized for optimal performance and that the concentrations of gates and input strands should be high enough (typically at the nanomolar level) to trigger the speedy strand displacement reactions [51]. However, this is not a significant problem, as the literature has already established strategies for optimizing toehold length and sequence composition and attaining fast displacement of strands [62]. It is important to note that there will be some natural limitations in terms of kinetics when using biomolecules in logic gate systems. The key steps that determine the kinetics of the DNA logic gate are the toehold displacement and strand dissociation reactions. Our data show that these steps occur quite rapidly initially as shown by the significant increase in the fluorescence intensity (two-three fold) within the first 30–60 s and that the fluorescence reaches the near-saturation level in ~2–3 min. The kinetics of strand displacement depends on the toehold length, the concentration of the input strand(s), and the gates themselves, and thus, it can be tuned by playing with these parameters—which is, in fact, an advantage of the DNA-based logic gates, as they offer a high level of design flexibility. Overall, given the low background, easily tunable, and simple operation of the logic gates demonstrated here, we envision that these DNA logic gates are suitable for the development of molecular circuits, as well as diagnostic tools, particularly for nucleic acid sensing, such as miRNA biomarkers and for multi-input analysis, and can provide building blocks for the development of biocompatible intelligent devices.

## Data Availability

Data are contained within the article and Supplementary Materials.

## Supplementary Materials

The following supporting information can be downloaded at https://www.mdpi.com/article/10.3390/nano14141185/s1, The Supporting Information includes sequences of the DNA oligonucleotides used in this study; the alternate logic gate designs and data; and gel-shift assay data.
