# Supplementary material for: Fluorescence-Based Multimodal DNA Logic Gates
Source: Nanomaterials (Basel). 2024 Jul 12;14(14):1185. doi: 10.3390/nano14141185 (PMC11280116; doi:10.3390/nano14141185)
Supplement: Supplementary file 1 [file nanomaterials-14-01185-s001.zip › nanomaterials-3087318-supplementary.pdf]

Supplementary Information

Fluorescence-Based Multimodal DNA Logic Gates

Chamika Harshani Algama, Jamil Basir, Kalani M. Wijesinghe, and Soma Dhakal*
Department of Chemistry, Virginia Commonwealth University, Richmond, VA 23284, USA
*Correspondence: sndhakal@vcu.edu

Table S1. DNA sequences used in the study.

Strand Name	Sequence (5'-3')
Strand A	GTG TGT TCC AAT CCC A
Strand B	TGG GAT TGT CTG TGT G/BHQ2/
Strand C	Cy3/CAC ACA GAG GTT AGG G
Strand D	CCC TAA CCG AAC ACA C
Strand E	GTG TGT TCG ACA CAT A
Strand A_1	GTG TGT TCC AAT CCC AGT CTA CCC
Strand A_2	CGT GAG TAG TGT GTT CCA ATC CCA
Strand B_2	TGG GAT TGT ATG TGT CTC TGT GTG /BHQ2/
Strand C_2	Cy3/CAC ACA GAG ACA CAT AGG TTA GGG
Strand D_1	TCC TCT CAC CCT AAC CGA ACA CAC
Strand D_2	CCC TAA CCG AAC ACA CAC TCT CCG
Input A_1*	GGG TAG ACT GGG ATT GGA ACA CAC
Input A_2*	TGG GAT TGG AAC ACA CTA CTC ACG
Input B_2 *	CAC ACA GAG ACA CAT ACA ATC CCA
Input C_2 *	CCC TAA CCT ATG TGT CTC TGT GTG
Input D_1*	GTG TGT TCG GTT AGG GTG AGA GGA
Input D_2*	CGG AGA GTG TGT GTT CGG TTA GGG
Strand F	CAA TCC CAA CTC TCC G
Strand G	GAA CAC ACG TCT ACC C

YES-1

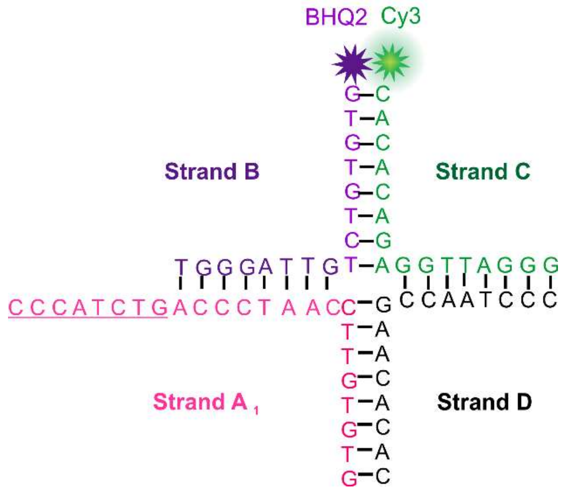

Figure S1. Four-way DNA junction (YES-1 gate) with the sequence detail and BHQ2 and Cy3 positions. The underlined portion of the strand A_1 represents toehold.

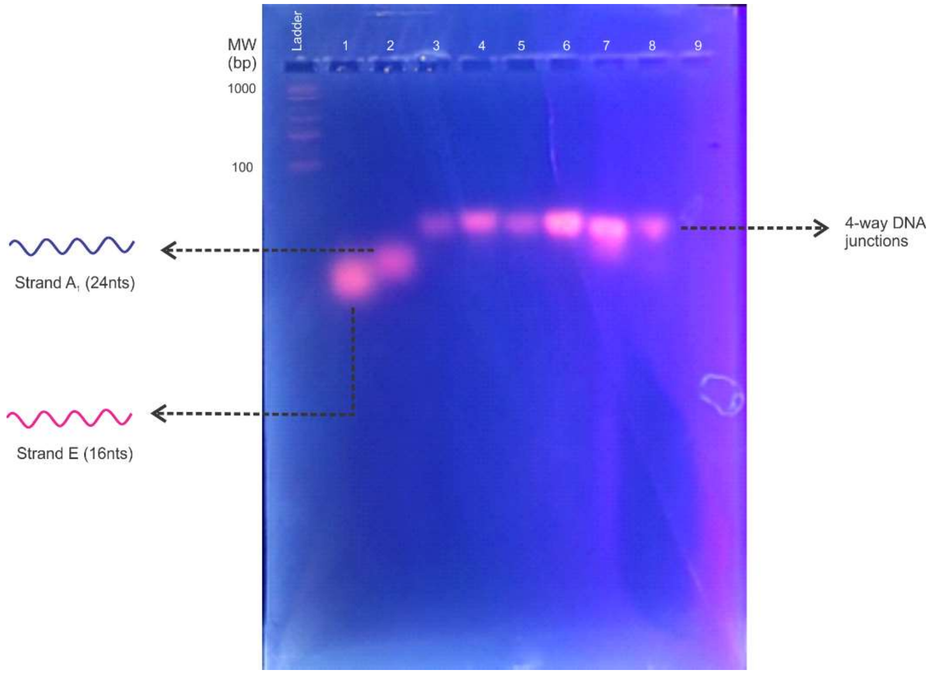

Figure S2. Agarose gel characterization of the formation of four-way DNA junctions using a native gel electrophoretic mobility shift assay (native-EMSA). Lane 1: DNA molecular weight (MW) marker. Lane 1: Strand E (16nts). Lane 2: Strand A_1 (24nts). Lane 3-5: YES-1, AND-1, and OR-1 respectively. Lane 6: Representative gate for simultaneous operation; AND-2. Lane 8 and 9: Unit 1 and Unit 2 for Cascading circuits. The slower migration of the bands in lanes 3-8

compared to lanes 1 and 2 indicates the successful assembly of the four-way DNA junction. The gel was stained in Gel Red before running for 60 minutes at 65V and visualized under a UV transilluminator.

Table S2. YES-1 gate standard deviation and explanation.

YES-1 gate (without Input)		YES-1 gate (with Input A_1*)	
Time (min)	Mean fluorescence (RFU)	Time (min)	Mean fluorescence (RFU)
0.5	13.8 ±0.7	0.5	96.4 ± 10
1	15.0 ±0.5	1	193.0 ± 10
1.5	16.8 ±0.8	1.5	261.5 ±7
2	19.7 ±0.4	2	306.2 ±6
2.5	22.4 ±0.7	2.5	333.6 ±2
3	23.8 ±0.6	3	353.5 ±2
3.5	27.0 ±0.7	3.5	366.9 ±1
4	28.6 ±0.9	4	378.4 ±1
4.5	30.7 ±0.4	4.5	386.4 ±0.7
5	32.9 ±0.6	5	393.1 ±2

The errors (represented by the standard deviations) are relatively higher initially – which we think is due to the discrepancy in data recording time from experiment to experiment. A small discrepancy in the measurement time can give a significant difference in the fluorescence readout of replicates in the fast kinetics regime (first two minutes). However, it is important to note that the error dropped to less than 1% when it gets to near saturation. These errors, in principle, can be reduced by automated measurements.

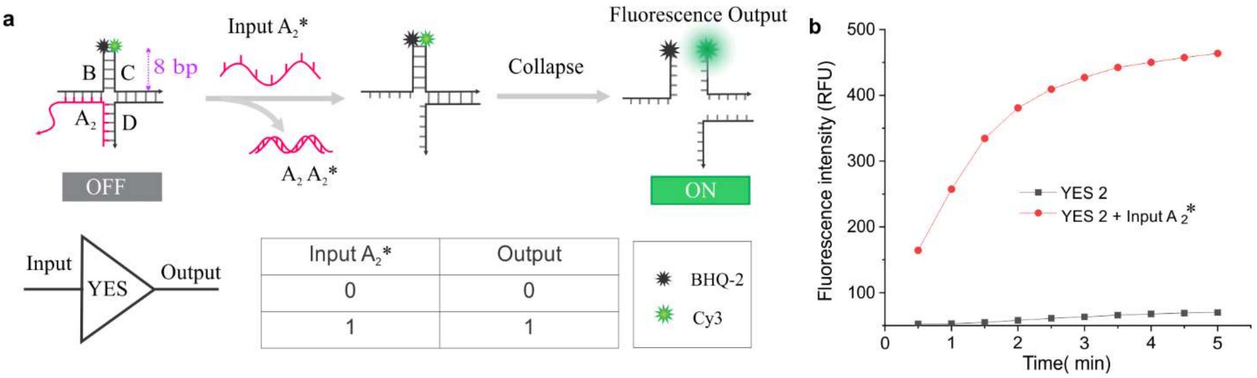

Figure S3. (a) Schematic illustration of the working principle of the YES-2 gate and the truth table with and without input. (b) Fluorescence signal in the absence (control) and the presence of input (A_2*) over time.

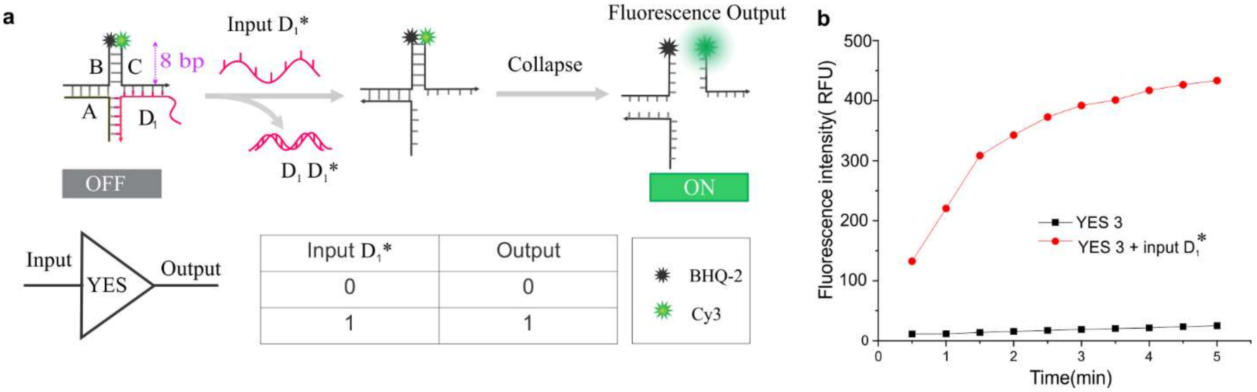

Figure S4. (a) Schematic illustration of the working principle of the YES-3 gate and the truth table with and without input. (b) Fluorescence signal in the absence (control) and the presence of input (D_1*) over time.

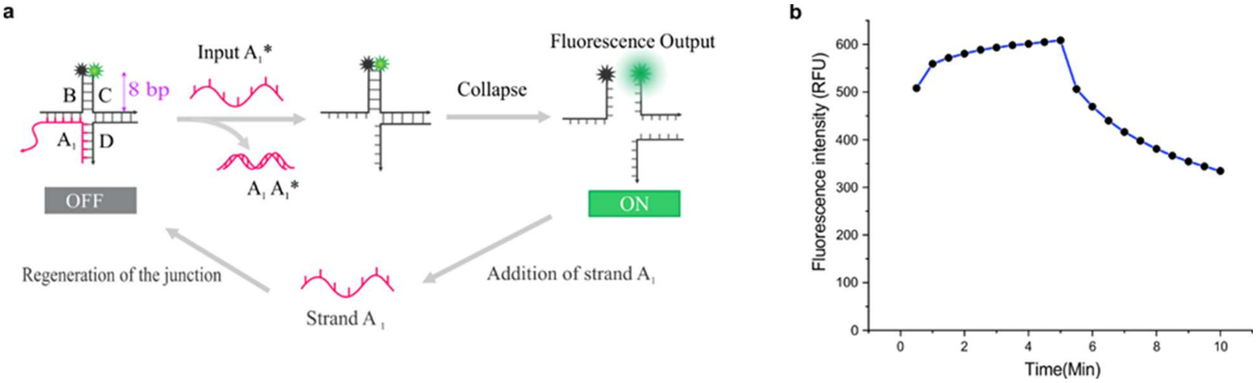

Figure S5. (a) Schematic illustration of the recyclability of YES-1 gate. (b) Fluorescence signal in the presence of input A1* and strand A1.

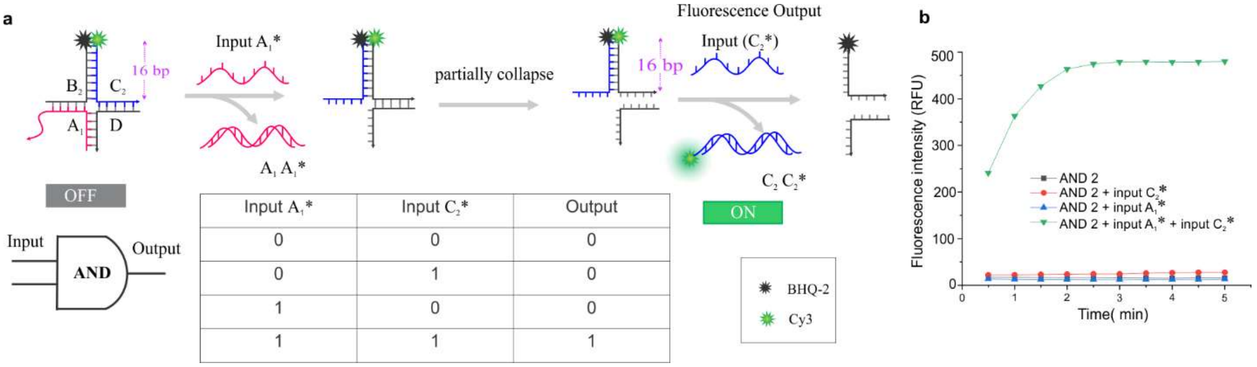

Input A_1*	Input C_2*	Output
0	0	0
0	1	0
1	0	0
1	1	1

Figure S6. (a) Schematic illustration of the working principle of the AND-2 gate and the truth table with and without input. (b) Fluorescence signal in the absence (control) and the presence of inputs (A_1* & C_2*) over time.

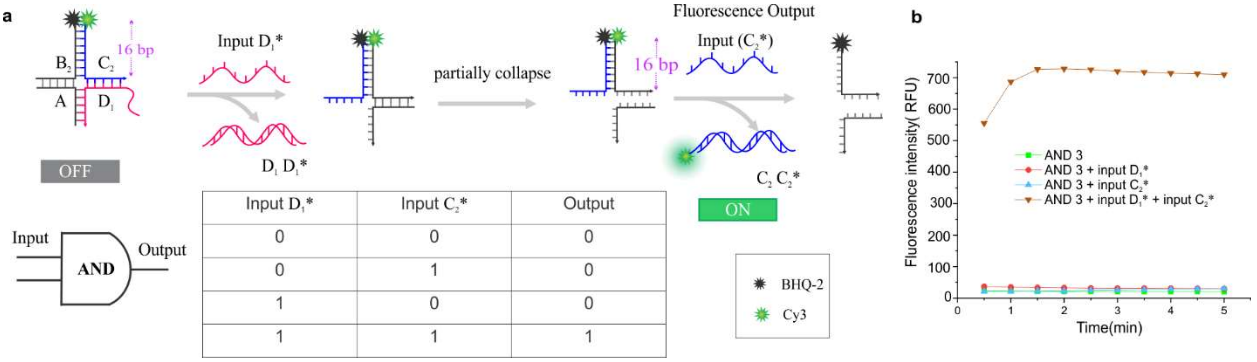

Input D_1*	Input C_2*	Output
0	0	0
0	1	0
1	0	0
1	1	1

Figure S7. (a) Schematic illustration of the working principle of the AND-3 gate and the truth table with and without input. (b) Fluorescence signal in the absence (control) and the presence of inputs (D_1* & C_2*) over time.

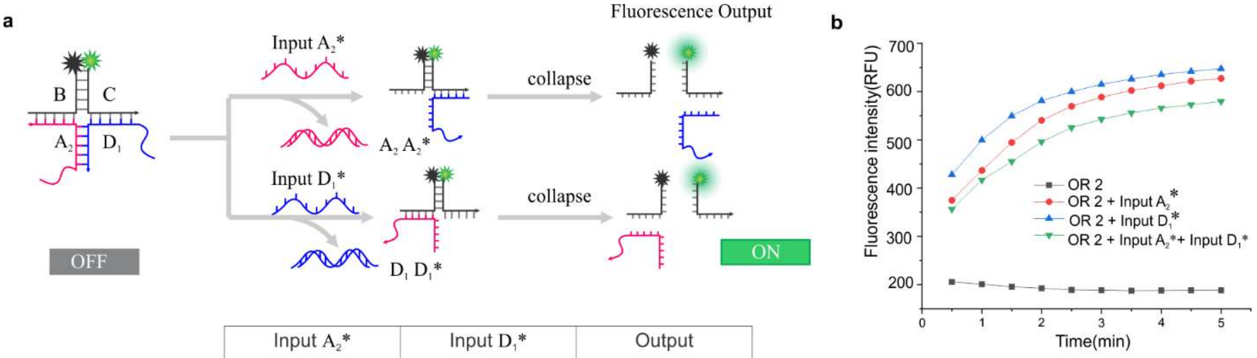

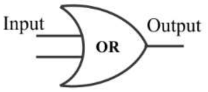

Input A_2*	Input D_1*	Output
0	0	0
0	1	1
1	0	1
1	1	1

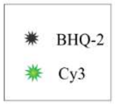

Figure S8. (a) Schematic illustration of the working principle of the OR-2 gate and the truth table with and without input. (b) Fluorescence signal in the absence (control) and the presence of inputs (A_2* & D_1*) over time.

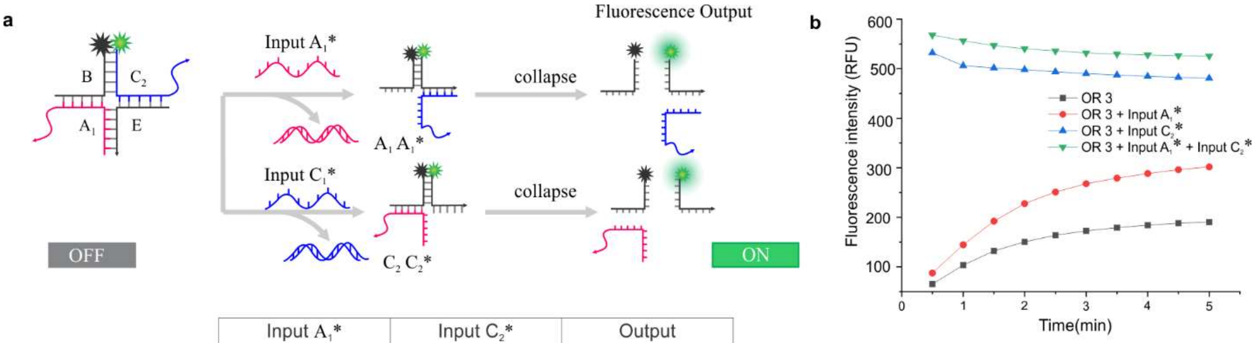

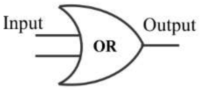

Input A_1*	Input C_2*	Output
0	0	0
0	1	1
1	0	1
1	1	1

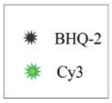

Figure S9. (a) Schematic illustration of the working principle of the OR-3 gate and the truth table with and without input. (b) Fluorescence signal in the absence (control) and the presence of inputs (A_1* & C_2*) over time.
